# Ultrasound Imaging: Advancing the Diagnosis of Periodontal Disease

**DOI:** 10.3390/dj13080349

**Published:** 2025-07-29

**Authors:** Gaël Y. Rochefort, Frédéric Denis, Matthieu Renaud

**Affiliations:** 1 Faculty of Odontology, Tours University, 37000 Tours, France; gael.rochefort@univ-tours.fr (G.Y.R.); frederic.denis@univ-tours.fr (F.D.); 2 Department of Medicine and Bucco-Dental Surgery, Tours University Hospital, 37044 Tours, France; 3 Bioengineering Biomodulation and Imaging of the Orofacial Sphere, 2BIOS, Odontology Department, Tours University, 37000 Tours, France; 4 N2Cox U1069 INSERM, Tours University, 37032 Tours, France; 5 EA 75-05 Education, Ethique, Santé, Faculté de Médecine, Université Franςois-Rabelais, 37044 Tours, France

**Keywords:** ultrasound, periodontitis, periodontal inflammation, artificial intelligence

## Abstract

**Objectives**: This pilot study evaluates the correlation between periodontal pocket depth (PPD) measurements obtained by manual probing and those derived from an AI-coupled ultrasound imaging device in periodontitis patients. **Materials and Methods**: Thirteen patients with periodontitis underwent ultrasonic probing with an AI engine for automated PPD measurements, followed by routine manual probing. **Results**: A total of 2088 manual and 1987 AI-based PPD measurements were collected. The mean PPD was 4.2 mm (range: 2–8 mm) for manual probing and 4.5 mm (range: 2–9 mm) for AI-based ultrasound, with a Pearson correlation coefficient of 0.68 (95% CI: 0.62–0.73). Discrepancies were noted in cases with inflammation or calculus. AI struggled to differentiate pocket depths in complex clinical scenarios. **Discussion**: Ultrasound imaging offers non-invasive, real-time visualization of periodontal structures, but AI accuracy requires further training to address image artifacts and clinical variability. **Conclusions**: The ultrasound device shows promise for non-invasive periodontal diagnostics but is not yet a direct alternative to manual probing. Further AI optimization and validation are needed. **Clinical Relevance**: This technology could enhance patient comfort and enable frequent monitoring, pending improvements in AI reliability.

## 1. Introduction

Periodontal disease is an inflammatory disease of multifactorial etiology [1]. It is characterized by progressive gingival inflammation with destruction of the tooth-supporting tissues (bone, alveolar ligaments), leading to tooth mobility that can lead to tooth loss [1]. Violation of sulcus by restorative margins in the supracrestal connective tissue or by the presence of biofilm is associated with inflammation and loss of periodontal attachment [2]. Periodontal health assessment is based on several clinical criteria, including sulcus depth and attachment loss. To measure it, the clinician uses a graduated periodontal probe. Pocket depth refers to the measurement from the gingival margin to the base of the periodontal pocket. Attachment level is defined as the distance from the cemento-enamel junction to the base of the periodontal pocket [3,4]. The probe has the particularity of being graduated and having a rounded end. Some are electronic and calibrated to allow better control of the pressure exerted by the practitioner and to improve reproducibility [5]. The measurement result depends on multiple aspects, such as the probe’s configuration, the thickness of the tip, the measurement angle, the pressure exerted, and the degree of inflammation. The force exerted during probing may differ from one practitioner to another or from one session to another. Furthermore, if the gingiva is inflamed, the tip of the probe can pass through the inflamed tissue, resulting in an overestimation of the pocket measurement [6]. These factors lead to difficulties in the reproducibility of measurements. In addition, measurement by periodontal probe, due to its intrusive aspect, is very uncomfortable for the patient [3]. Under these conditions, the practitioner may inaccurately assess the pocket depth and may underestimate the measurement. It should also be noted that bacteria from a periodontal pocket can spread to a healthy tooth’s sulcus during the examination. This process is known as cross-contamination. This examination, which is unpleasant for the patient, lasts about half an hour but remains the gold standard for assessing the stage of periodontal disease in the absence of other methods [6].

Since the mid-1980s, numerous studies have reported on the use of ultrasound imaging to investigate the oral cavity in animals [7]. While previous studies, including our own [8,9], have explored intraoral ultrasonography for visualizing periodontal structures, this pilot study uniquely investigates the integration of artificial intelligence for automated periodontal pocket depth (PPD) measurements, comparing these with manual probing to assess clinical feasibility and accuracy. Ultrasonography of the periodontal tissues could constitute an alternative for the visualization of the periodontal structures. Ultrasound-based assessment of periodontal pocket depth and identification of inflammation in periodontal tissues may support earlier detection and management of periodontal disease. In recent years, the emergence of artificial intelligence has enabled the development of tools to assist humans in improving diagnosis, assessing risks, and improving disease management. Artificial intelligence (AI) offers new possibilities to address these challenges by exploiting machine learning algorithms and big data analysis to improve decision-making processes. Several studies have explored the use of AI algorithms for the automated detection of periodontal diseases from intraoral photographs and radiographs (retro-alveolar radiograph, dental panoramic) with results that suggest that AI-based systems have the potential to help clinicians detect and diagnose periodontal diseases early [10]. Many AI-based machine learning models have been shown to perform as well as highly trained physicians. Moreover, although many predictive tasks performed by AI replicate those typically performed by human experts, some models are capable of uncovering diseases and patient features that go beyond the original purpose of the imaging techniques used [11]. A recent narrative review highlighted that incorporating artificial intelligence into personalized diagnostics in periodontology could transform the field by enhancing diagnostic accuracy and efficiency, enabling tailored treatment strategies, and improving patient care outcomes [12].

Our research teams are developing an intraoral ultrasound probe specifically designed for examining periodontal tissues. This tool provides high-resolution real-time imaging of the human periodontium and is equipped with AI technology to automatically measure periodontal pocket depth. In this context, the prospect of a new diagnostic tool for periodontal diseases using high-resolution ultrasound imaging coupled with AI seems to emerge for direct and automatic detection and measurement of periodontal pockets. The objectives of this prospective pilot study were to evaluate the correlation between periodontal pocket depth measurement obtained by periodontal probing and periodontal pocket depth measurement obtained by AI via the ultrasound imaging device.

## 2. Materials and Methods

This pilot study is a prospective, single-center clinical trial. Thirteen patients with initial periodontitis in need of periodontal treatment were recruited. A special assessment was performed by ultrasonic probing coupled with an AI engine in order to obtain the measurements automatically. Following this examination, a routine periodontal assessment was performed on each patient with a periodontal probe with 1 mm gradations (OMS type). The assessments have been performed in the service of oral medicine and surgery at Tours Hospital (Tours, France). This study has been approved by the Comité de Protection des Personnes Est 1 (n°22.04642.000161, approved on 9 February 2023) and registered in the International Clinical Trials Registry Platform (ICTRP) under the ID: NCT05809427.

### 2.1. Inclusion Criteria

Patient:

1. ≥18 years of age
2. Participants covered by national health insurance or equivalent
3. Written informed consent obtained from the participant or participant’s legal representative
4. Ability for participant to comply with the requirements of the study

Teeth:

1. Minimum of 14 teeth
2. Presence of at least one tooth with a pathological periodontal pocket (≥4 mm).

### 2.2. Exclusion Criteria

1. Surgical procedure performed in the area to be scanned
2. Osteosynthesis material
3. Patients under legal guardianship or judicial protection
4. Pregnancy, breastfeeding
5. Inclusion in another therapeutic trial

### 2.3. Ultrasound Device

**Figure 1:**
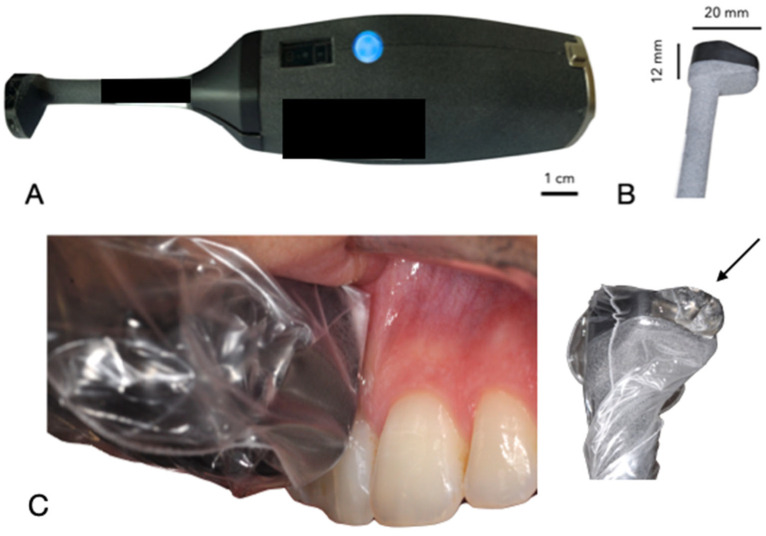
Intraoral ultrasound probe. (**A**) Probe design with ergonomic handle and (**B**) transducer head. (**C**) Intraoral positioning parallel to the tooth’s long axis, associated with a specialized intraoral coupling gel (arrow).

The ultrasound device, developed by Carestream Dental (Atlanta, GA, USA) in collaboration with Tours University Hospital and the GREMAN UMR 7347 research consortium, is a compact, wireless intraoral probe (dimensions: 15 cm length, 2 cm diameter at the handle, 1 cm at the probe head) designed for periodontal tissue examination [9]. Its ergonomic design facilitates easy handling within the oral cavity, with a lightweight structure (approximately 150 g) and a disposable hygienic cover. The device uses a 20 MHz high-frequency transducer, achieving an axial and lateral resolution of approximately 100 µm. The data are transmitted via Wi-Fi to a connected computer, where AI algorithms were trained to measure in real-time PPD based on anatomical landmarks (e.g., epithelial-connective tissue attachment, gingival margin). However, it faced challenges in distinguishing pocket depths in cases with inflammation or calculus due to ultrasound artifacts like drop shadows. Figure 1 illustrates the probe’s design and intraoral positioning.

### 2.4. Sulcus Depth Measurement

Sulcus depth was measured by manual probing (OMS-type) by the same periodontal specialist with a millimeter probe (gold standard) along the tooth and in its axis, exerting a pressure of 0.25 N (equivalent to 25 g). Three measurements per tooth and per side (vestibular and lingual) were performed (Mesial, Central, Distal) on all teeth present on each patient.

### 2.5. Interventions

Only one visit was planned per patient, during which the adverse events and possible defects of the experimental medical device were explained. The manual probe was part of routine care and did not constitute an act specifically added by the clinical investigation.

The measurement with the ultrasound probe was carried out before the measurement by manual probing to avoid that the introduction of the metal probe into the periodontal space would cause lesions of the gum, impacting the measurements obtained by the ultrasound device (if carried out afterwards).

The clinical investigation was carried out as follows:

1. Information and signing of consent for the clinical study.
2. Data Collection with the investigational device:
  ▪ Automatic periodontal measurements performance using the embedded artificial intelligence module;
  ▪ Manual Periodontal probing using probe.

**Figure 2:**
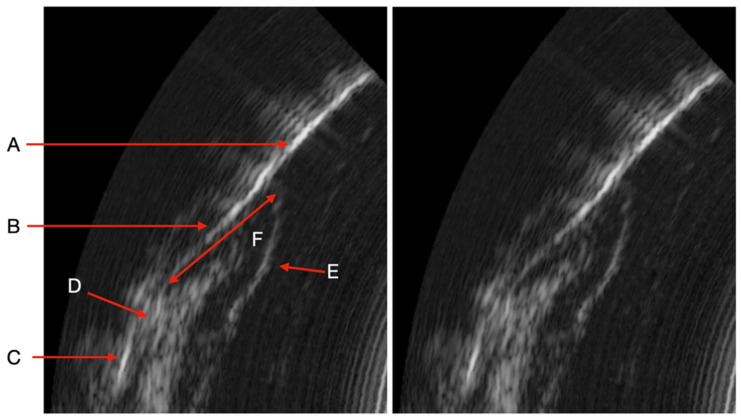
Image obtained by the US device (A: Enamel. B: Cemento-enamel junction. C: Alveolar bone crest. D: Connective tissue attachment. E: Keratinized free gum. F: Sulcus/pocket area).

For ultrasound imaging measurement, the patient was positioned comfortably in a dental chair in a semi-upright posture. The ultrasound probe, which is smaller than standard intraoral optical cameras, facilitates access to all areas of the oral cavity. A disposable cover was placed over the probe head for hygiene. With the patient’s mouth partially open to allow gentle, unobstructed access, the operator carefully introduced the probe into the oral cavity. A specialized intraoral coupling gel (AEVO Oral Ultrasound Gel, SmileSonica Inc., Edmonton, Canada) was applied between the probe head and the tissue to ensure effective ultrasound transmission from the emitter to the tissue (Figure 1). The probe head was aligned both perpendicularly and parallel to the targeted tissue, using the tooth’s long axis as a reference parallel to the probe’s axis. The scanning surface of the probe was kept as perpendicular as possible to the soft tissue to prevent distortion (Figure 2). For operational convenience, scanning started with the buccal surfaces of the upper and lower arches, proceeding in a right-to-left and forward-backward motion—from tooth 17 to 27, then from 47 to 37. Each soft tissue area was scanned for approximately 2–3 s, covering one tooth between its mesial and distal contacts. The same process was then applied to the lingual surfaces, following the same scanning sequence. The probe transmitted data via Wi-Fi to a connected computer, where AI-powered automatic measurements were displayed in real time and organized according to the standard dental charting system.

At the end of the investigation, the participants also completed a satisfaction questionnaire. The total visit duration during the study was 60 min.

### 2.6. Study Flow

**Figure 3:**
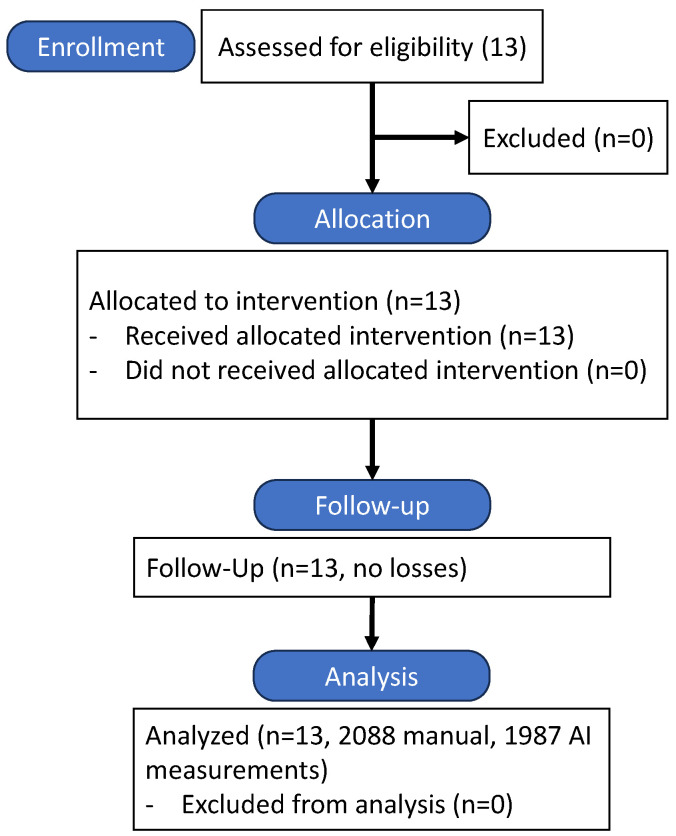
CONSORT flow diagram of participant recruitment and study flow.

The study followed a prospective, single-center design, with participant recruitment and flow detailed in Figure 3. Thirteen patients meeting inclusion criteria were enrolled, and all completed the study protocol without dropouts.

### 2.7. Statistical Analysis

The primary outcome was the correlation between PPD measured by manual probing and AI-based ultrasound imaging, assessed using a Pearson correlation coefficient. The 95% confidence interval was calculated using the bootstrap method with the participant as the unit of resampling. Descriptive statistics (mean, range, and pocket depth categories) were computed for both methods.

## 3. Results

A total of 13 subjects were included (5 women, 8 men; mean age 53 years). The median duration of manual probing was 6 min (IQR: 5–6 min), while ultrasound probing took 19 min (IQR: 16–21 min). A total of 2088 manual probing measurements and 1987 AI-based ultrasound measurements were collected. Table 1 summarizes PPD measurements. The mean PPD was 2.95 ± 1.38 mm (range: 1–10 mm) for manual probing and 3.04 ± 1.48 mm (range: 0–9 mm) for AI-based ultrasound, with a weak Pearson correlation coefficient of 0.033 (*p* = 0.144) and Lin’s concordance coefficient was 0.033. The mean difference was −0.09 mm, with limits of agreement from −3.99 mm to +3.81 mm.

The exact concordances between stratified pocket depth measures are weak and not significant: pockets < 3 mm, n = 878, exact concordance of 18.7%; pocket 3–5 mm, n = 1015, exact concordance of 23.3%; pocket > 5 mm, n = 94, exact concordance of 3.2%. However, concordance within ±1 mm reached 61.5% in 3–5 mm pockets (*p* = 0.022), but only 10.6% in >5 mm pockets (Table 2). Discrepancies were observed in cases with deep inflammation or calculus, where AI struggled to differentiate pocket depths from surrounding structures.

The ultrasound (US) probe captures real-time images (US probing). All periodontal structures are clearly visible in the image under physiological and pathological conditions. Periodontal tissues (enamel, cemento-enamel junction, alveolar bone crest, connective tissue attachment, keratinized free gum, sulcus/pocket area) can be easily identified after a short period of learning to read ultrasound images. These structures need to be identified so that the AI algorithm can then automatically measure the sulcus area. All periodontal structures appear in Figure 2.

**Figure 4:**
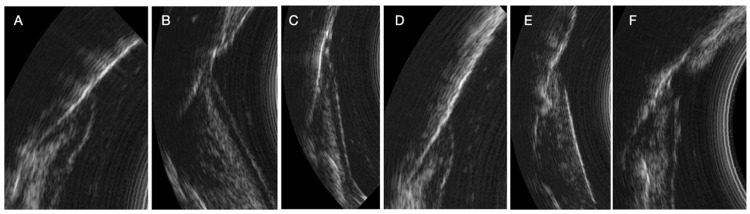
Variations in periodontal ultrasound images. (**A**) Reference image with normal structures. (**B**) Presence of deep inflammation with visible connective tissue junction. (**C**) Presence of deep inflammation without visible connective tissue junction. (**D**) Presence of supragingival calculus. (**E**) Presence of subgingival calculus. (**F**) Presence of calculus above and below the gingival surface and deep inflammation.

During the study, we observed many variations in ultrasound images depending on the clinical situation (inflammatory area, presence of visible calculus, edema, spontaneous bleeding, normal situation). Indeed, the images can reveal the presence of inflammation in the deep gingival tissues. Sub-gingival calculus can also be visible. The image quality is also different because of the variation in the ultrasound signal [13]. The same is true for subgingival calculus which can also be objectified on the ultrasound images. Although many of these elements appear in the image, AI currently struggles to differentiate pocket depths from surrounding structures in certain clinical contexts. These structures need to be identified and characterized for more precise training of the AI (Figure 4)

## 4. Discussion

The AI algorithm in this study was trained to measure PPD using anatomical landmarks such as the epithelial-connective tissue attachment and gingival margin. However, inconsistencies between manual and AI-based measurements (Table 1) were observed, particularly in cases with inflammation or calculus, due to ultrasound artifacts like drop shadows [14]. Future in vitro studies correlating ultrasound signals with histological structures are needed to validate these reference points and improve AI accuracy.

The advantage of a measurement carried out automatically on an image is the possibility of obtaining a real clinical measurement in relation to points that are histological references [14,15]. When manual probing considers a feeling of pressure of the probe in the sulcus and a reading at the level of the free gingiva, AI considers measurements in relation to real tissue references: epithelial-connective tissue attachment and top of the free gingiva. The advantage of having a real-time image exploited and analyzed by AI is the detection of elements that can generate a manual measurement bias such as subgingival calculus for example, or significant inflammation of the tissues. AI could make it possible to indicate or even quantify the presence of subgingival calculus and inflammation for the diagnosis or monitoring of periodontal treatments.

However, there are many inconsistencies between manual periodontal pocket measurements and measurements made by US and IA probing. At this stage, many challenges remain to be overcome. To begin with, ultrasound imagery produces artifacts that are unique such as the “drop shadow”. This is the fact that a structure always appears less echogenic (blacker) than it really is when it is preceded (on the path of the ultrasound) by a hyperechoic structure [14]. In the next phase, developing a new AI model involves a team of experts who thoroughly review the entire dataset to inform the model’s design. This step is essential, as the dataset may contain inherent biases, confounding variables, or underlying risk factors that must be accounted for. This requires steps to modify the model or the dataset on which the model was trained to ensure accuracy and limit measurement bias [15,16,17].

It should be noted that we can observe several clinical situations that can give a bias in measuring the periodontal pocket automatically by AI (Figure 2). The detection of significant inflammation in the image can greatly influence the outcome in the difference in pocket depth measurements obtained with the AI algorithm and the manual probe. Indeed, there are situations where the boundary between the inflammation image and the bottom of the pocket is not clear. This is explained by the absence of connective tissue visible on the image. In these specific cases, as it is trained today, the AI algorithm measures the depth of the periodontal pocket associated with the amount of inflammation. In other situations, the boundary is clearly visible on the image and the distinction between the periodontal pocket and the inflammation is easily identifiable by AI. This type of image alerts the clinician to the reality of the pocket. Indeed, can we say whether the pocket measured with the manual probe is really the one to be considered if, in addition, a deep inflammatory zone is already present on the ultrasound imaging. In addition, other images show the presence of supra or subgingival calculus. The same is true for the presence of deep calculus, at the bottom of the periodontal pocket, which can impact the measurement obtained by the AI algorithm of the area (current AI algorithm training specifications). Although the presence of tartar does not represent a diagnostic element in the determination of periodontal disease, the possibility of being able to identify the presence of tartar from the initial clinical examination (to choose the scaling strategy), at the end of the instrumentation (to evaluate the effectiveness of the procedure), as well as during the maintenance phases, represents a considerable impact in terms of saving time, procedures, costs and, above all, reducing fatigue and wear of the dentist.

In more extreme situations combining deep inflammation and supra and subgingival tartar, the AI in its current configuration is unable to provide matching measurements as the conventional manual probing. The longer duration of ultrasound probing (median 19 min vs. 6 min for manual probing) and challenges in structure identification due to artifacts highlight areas for improvement. However, the contribution of AI is a real advance in bringing periodontal diagnosis closer to histological reality. Apart from the evaluation of the bleeding index during probing, it is currently impossible to know the real inflammatory phase of periodontal inflammation or to observe an early phase of healing or a gingival reaction to the oral environment. The current ultrasound device focuses on soft tissue and PPD measurements, limiting its ability to assess alveolar bone loss, which is better evaluated with modalities like cone-beam computed tomography (CBCT). Future integration with CBCT could provide a comprehensive periodontal assessment. Additionally, as the device is in development, cost-effectiveness studies are pending commercialization, but its portability and lack of ionizing radiation suggest potential economic benefits for routine practice.

All these elements are therefore measurement biases but can be corrected by more significant training of the AI algorithm (adapted rules for ground truth annotations). A debate must also clarify the choice of measurement by AI when there is deep inflammation. Should we take it into account? What about the state of the connective tissue attachment at this time of the examination? Establishing a specific score to report these elements to the clinician (subgingival calculus, pocket depth, deep inflammation) can also be an element of aid for more precise and early diagnosis, improving patient care [12].

Ultrasonic imaging provides a new perspective on the reality of periodontal structures at the time of clinical examination, which was not possible with traditional examination in the past [18,19,20]. A reflection on the reality of the periodontal pocket must be conducted by considering the presence of deep inflammation in the tissues to bring its histological state closer to its clinical state in order to better characterize the state of the pathology (acute, chronic, early, etc.) [18].

Compared to other diagnostic imaging modalities used in periodontology, such as conventional radiography, cone-beam computed tomography (CBCT), and magnetic resonance imaging (MRI), intraoral ultrasound imaging presents a unique combination of advantages. Traditional radiographic techniques, including periapical and panoramic radiographs, are limited to two-dimensional representations and cannot directly visualize soft tissue structures or assess periodontal inflammation. CBCT, while offering three-dimensional imaging, exposes the patient to ionizing radiation, is more costly, and remains less accessible in routine clinical settings. MRI provides excellent soft tissue contrast without radiation, but it is not commonly used in dentistry due to its high cost, limited availability, extended acquisition time, and patient discomfort related to the scanning environment. In contrast, intraoral ultrasound offers real-time imaging of both hard and soft periodontal tissues without ionizing radiation, at a significantly lower cost and with higher accessibility. The examination can be performed chairside in a matter of minutes and does not require complex infrastructure. Its portability, patient comfort, and compatibility with artificial intelligence for automated analysis make it a promising tool for daily clinical practice and for longitudinal monitoring of periodontal health. While further optimization and validation are needed, ultrasound imaging may represent a balanced compromise between diagnostic precision, safety, and clinical feasibility, especially when enhanced by AI-based interpretation.

The integration of artificial intelligence into real-time ultrasound imaging is expected to transform the standard of care in periodontal diagnostics. Beyond pocket depth measurement, AI models could be trained to detect additional clinical features such as bone resorption patterns, tissue elasticity, and early inflammatory markers that are difficult to capture with conventional methods. Such advancements could allow clinicians to not only quantify disease severity but also predict its progression, potentially enabling risk stratification and individualized treatment protocols. Moreover, combining ultrasound data with other patient-specific variables through multimodal AI approaches could open the door to precision periodontology. These developments mirror trends observed in other medical specialties, such as cardiology and dermatology, where AI-enhanced imaging has already improved diagnostic accuracy and workflow efficiency [21,22].

Despite its promising capabilities, the current implementation of AI in periodontal ultrasound imaging still faces several challenges. One of the main limitations lies in the variability of image acquisition due to operator-dependent factors and anatomical variability across patients. Additionally, the limited size and diversity of annotated training datasets hinder the generalizability of AI models. Future studies should focus on multicenter data collection, the standardization of annotation protocols, and the integration of clinical metadata to improve model robustness. Clinical trials assessing the longitudinal use of AI-ultrasound tools in daily practice are also necessary to evaluate their true clinical utility and cost-effectiveness. Regulatory frameworks and ethical considerations, particularly regarding data privacy and decision transparency, must also be addressed to facilitate clinical implementation [23,24,25].

This pilot study has several limitations. The small sample size (13 patients) and single-center design limit generalizability. Variability in ultrasound image acquisition due to operator technique and anatomical differences affected AI performance. The AI algorithm struggled with complex clinical scenarios (e.g., inflammation, calculus), highlighting the need for larger, annotated datasets and improved training protocols. Future research should include multicenter trials, in vitro validation, and longitudinal studies to assess clinical utility and cost-effectiveness.

## 5. Conclusions

The intraoral ultrasound device, coupled with AI, demonstrates potential for advancing periodontal diagnostics by providing non-invasive, real-time imaging of periodontal structures (Table 2). However, current limitations, including discrepancies in AI-based PPD measurements compared to manual probing (Table 1), indicate that it is not yet a direct alternative to conventional methods. Advantages include patient comfort, absence of ionizing radiation, and potential for reduced bacterial dissemination. Further optimization of AI algorithms, addressing image artifacts and clinical variability, is essential. Future studies should focus on larger datasets, in vitro validation, and exploring additional metrics like inflammation indices to enhance diagnostic precision.

## Figures and Tables

**Table 1:** Comparison of Periodontal Pocket Depth (PPD) measurements.

Measurement Method	Number of Pockets	Mean PPD ± SD (mm)	Range (mm)
Manual Probing	2088	2.95 ± 1.38	1–10
AI-Based Ultrasound	1987	3.04 ± 1.48	0–9

**Table 2:** Concordance within ± mm in stratified pocket by depth ranges.

Class	Number of Pockets	Concordance ± 1 mm (%)	Pearson r	*p*-Value
**<3 mm**	878	57.7	0.016	0.643
**3–5 mm**	1015	61.5	0.072	0.022
**>5 mm**	95	10.6	0.016	0.878
